# Recanalization of atherosclerotic stenosis and occlusion of intracranial vertebrobasilar artery

**DOI:** 10.1016/j.ibneur.2024.12.016

**Published:** 2024-12-31

**Authors:** Zhi-Long Zhou, Liang-Fu Zhu, Tian-Xiao Li, Bu-Lang Gao

**Affiliations:** Stroke Center, People's Hospital of Zhengzhou University, Henan Provincial People’s Hospital, School of Clinical Medicine, Henan University, 7 Weiwu Road, Zhengzhou, Henan 450003, China

**Keywords:** Intracranial vertebrobasilar atherosclerotic stenosis, Posterior circulation, Endovascular recanalization, Angioplasty, Stenting

## Abstract

Intracranial vertebrobasilar atherosclerosis is one of the major causes of posterior circulation ischemic strokes and may result in a high risk of recurrent stroke. Current treatment for vertebrobasilar stenosis comprises aggressive medication and percutaneous transluminal angioplasty and stenting (PTAS). Endarterectomy and intracranial-extracranial bypass surgery may be considered for large artery stenosis in the anterior circulation, but they may be deterred by some technical difficulties and great complication rates in the vertebrobasilar stenoses. PTAS may be good for eliminating the arterial stenosis and preventing recoil of arterial wall after balloon angioplasty alone, however, higher peri-procedural complications and poor follow-up outcomes prevent PTAS as a common treatment strategy. Nonetheless, for selected patients with severe (≥70 %) stenosis and non-acute occlusion of the intracranial vertebrobasilar artery refractory to the best medication, PTAS may be feasible, safe and effective if the treatment approaches and endovascular devices were tailored to the clinic-radiological features of each lesion and patient. Sub-satisfactory stenting recanalization of the stenosis using a balloon < 80 % of the diameter of the nearby normal artery for dilatation of the stenosis and selection of softer and more flexible stents and delivery systems may be advantageous in decreasing the peri-procedural complications. This study reviewed the concept of intracranial vertebrobasilar atherosclerotic stenosis, available treatment modalities, peri-procedural complications, risk factors for endovascular treatment and prognosis, evidence for sub-satisfactory recanalization of the stenosis, and strategies to improve the peri-procedural complications and prognosis with the hope of improving the treatment outcome of endovascular recanalization.

## 1 Introduction

Approximately 20 %-25 % of all cerebral ischemic strokes are caused by posterior circulation ischemia, with a 1-month death rate ranging 3.6 %-11 % (Caplan et al., 2004, Dewey et al., 2003, Palmisciano et al., 2023). One of the major causes of posterior circulation ischemia is intracranial atherosclerosis which may lead to higher risks of recurrent ischemia in vertebrobasilar stenoses in comparison to those of anterior circulation ischemia in carotid stenoses (Marquardt et al., 2009). Vertebrobasilar stenosis may cause cerebral hypoperfusion and subsequent transient or permanent neurological impairment like transient ischemic attack and stroke (Zhang et al., 2020, Reith et al., 2015). Current treatment for vertebrobasilar stenosis comprises aggressive medication, including dual antiplatelet therapy and risk factor control, and percutaneous transluminal angioplasty and stenting (PTAS). Endarterectomy and intracranial-extracranial bypass surgery may be considered for large artery stenosis in the anterior circulation, but they may be deterred by some technical difficulties and great complication rates in the vertebrobasilar stenoses. Two recent important clinical trials of SAMMPRIS and VISSIT (Chimowitz et al., 2011, Zaidat et al., 2015) compared the effect and safety between medical treatment and PTAS and disfavored the use of PTAS because of a higher rate of 30-day recurrent strokes after PTAS compared to aggressive medical treatment. In spite of this outcome for PTAS, the poor natural history of vertebrobasilar stenosis usually necessitates endovascular recanalization for medical-refractory patients in order to prevent long-term neurological impairment because intensive medical therapy alone cannot exempt high-risk recurrent strokes (Chimowitz et al., 2005, Mazighi et al., 2006). In the Warfarin-aspirin symptomatic intracranial disease (WASID) clinical trial (Chimowitz et al., 2005), patients with a stenosis greater than 50 % still had a 1-year recurrent rate of ischemic stroke up to 15 % in the aspirin arm and 14 % in the warfarin arm, with a respective 2-year recurrent rate of 20.4 % and 17 %. In the GESICA prospective study of symptomatic atherothrombotic intracranial stenosis (Mazighi et al., 2006), the two-year rate of recurrent stroke of patients with symptomatic intracranial stenosis treated with intensive medication was 38.2 %. Moreover, recently-published investigations have presented improved outcomes of PTAS with the Wingspan stent for intracranial atherosclerotic stenosis, resulting in a 2.6 % periprocedural stroke, bleeding, and mortality rate (Alexander et al., 2019) and a 1-year stroke or mortality rate of 8.5 % (Alexander et al., 2021). The CASSISS study has also reported comparable effects of PTAS against medication alone, with a 30-day incidence of stroke or death of 5.1 % in the PTAS and 2.2 % in the medication group, but no significant (P > 0.05) differences in the mortality during 1-year follow-up (8.0 % in the PTAS vs. 7.2 % in the medication alone group) (Gao et al., 2022). While the American Heart Association Council on Cardiovascular Radiology and Intervention and Stroke Council has recommended intensive medication as the major treatment approach for patients with symptomatic intracranial atherosclerosis (Eskey et al., 2018), further intracranial endovascular treatment is also suggested for “proper” patients with severe stenosis, poor collateral circulation, or hypoperfusion ineffective to medication treatment. In this study, we aimed to review the effect, safety, and long-term prognosis of PTAS for atherosclerotic large vessel stenosis or occlusion (LVSO) in the posterior circulation so as to find measures to decrease the peri-procedural complications of angioplasty and stent deployment.

## 2 Mechanism of LVSO-related ischemic stroke

Intracranial atherosclerotic stenosis is a progressive condition characterized by accumulation of lipids and fibrous components in the walls of intracranial arteries and may result in mild arterial wall thickening or even significant luminal stenosis affecting the hemodynamics (Kim and Bonovich, 2014, Kang and Yoon, 2019). Based on the “response to injury theory”, the earliest events in atherosclerosis can be initiated by some insults such as physical injury caused by turbulent blood flow, increased blood pressure, hyperglycemia or hyperlipidemia (Kang and Yoon, 2019, Ross, 1995). After endothelial dysfunction proceeds to formation of intimal lipid layers or fatty streaks, leukocytes and smooth muscle cells migrate into the arterial wall before ultimate degradation of the extracellular matrix and plaque formation. The intracranial arteries being mostly involved by atherosclerosis are the middle cerebral artery in the proximal and middle segments and basilar artery followed by the supraclinoid internal carotid artery and intracranial vertebral artery (Chimowitz et al., 2011, Chimowitz et al., 2005).

Intracranial atherosclerotic stenosis may cause acute ischemic stroke via in situ thrombotic occlusion, hypoperfusion, artery-to-artery embolism, perforating vessel occlusion caused by plaque extension to the arterial ostia, or a combination of these factors (Kang and Yoon, 2019, Holmstedt et al., 2013). Of all these mechanisms, large vessel occlusion is typically associated with in situ thrombotic occlusion caused by unstable plaque rupture in advanced intracranial atherosclerotic stenosis (Kang and Yoon, 2019, Bang, 2014). Neuroimaging may have characteristic presentations for these mechanisms. Ischemic infarction in a watershed distribution on cerebral imaging may indicate hemodynamic compromise or hypoperfusion via a stenotic artery while a distal wedge-like territorial infarction may suggest artery-to-artery embolism (Holmstedt et al., 2013). High-resolution magnetic resonance imaging may be able to identify plaque extension over the ostia of a small perforating artery, which may cause lacunar infarctions.

## 3 Types of lesions and treatment

### 3.1 Intracranial vertebral artery stenosis

#### 3.1.1 Anatomy of the vertebral artery

The vertebral artery is typically divided into four segments anatomically: V1-V4 (Burle et al., 2022). Segments V1 to V3 start from the origin on the subclavian artery to where the vertebral artery pierces the dura mater at the foramen magnum level, and these segments are classified as the extracranial vertebral artery. Once the artery enters the dura mater, it becomes the intracranial vertebral artery segment, namely the V4 segment (Burle et al., 2022), which ranges from where the artery enters the dura mater to the position it joins the contralateral vertebral artery before forming the basilar artery. At this segment, there are two major branches: the anterior spinal artery and the posterior inferior cerebellar artery. The vertebral artery may be tortuous, especially at the initial V1 segment originating from the subclavian artery, and this tortuosity makes it a preferable location for various pathologies difficult to pass through by endovascular devices (Burle et al., 2022).

#### 3.1.2 Treatment approaches

The tortuous course and frequent formation of atherosclerotic stenosis and calcification of the extracranial and intracranial vertebral artery (Chimowitz et al., 2011, Chimowitz et al., 2005, Madonis and Jenkins, 2021) pose some technical challenges of endovascular treatment with significant risks of periprocedural complications. For patients diagnosed with atherosclerotic stenosis of the vertebral artery, antiplatelet medications are recommended, including aspirin, aspirin with modified-release dipyridamole, clopidogrel, or ticlopidine to prevent myocardial infarction, stroke, and transient ischemic attack (Naylor et al., 2018, Aboyans et al., 2018, Brott et al., 2011). Surgical revascularization includes endarterectomy and reconstruction surgery, which may be applied in the initial vertebral artery segments (Burle et al., 2022, Madonis and Jenkins, 2021). Although endovascular stent angioplasty has been widely performed as a promising choice for atherosclerotic stenosis of the extracranial vertebral artery (Stayman et al., 2011, Antoniou et al., 2012), both the VAST (Compter et al., 2015) and VIST (Markus et al., 2017, Markus et al., 2019) clinical trials have failed to confirm the superiority of stent angioplasty in preventing recurrent strokes in symptomatic patients compared to the best medical treatment. For intracranial vertebrobasilar stenosis, heterogenous outcomes have been presented by different studies (Eberhardt et al., 2006, Zhou et al., 2023a), with a high endovascular treatment risk in one study (Eberhardt et al., 2006) but reduced peri-procedural complications in the other (Zhou et al., 2023a). In the study investigating the safety and efficacy of the Enterprise stenting for symptomatic atherosclerotic severe (≥70 %) posterior circulation stenosis enrolling 103 patients with intracranial vertebral artery stenosis and 159 patients with basilar artery stenosis (Zhou et al., 2023a), peri-procedural complications occurred in 14 (5.3 %) patients, with recurrent ischemic symptoms in 7 patients (2.9 %) and in-stent restenosis in 35 patients (15.7 %) at 1-year follow-up even though the restenosis rate was significantly greater in the intracranial vertebral artery than in the basilar artery (26.4 % vs. 8.8 %, P < 0.001). These good outcomes may be partially attributed to the better performance of the more flexible Enterprise stent which has a smaller radial force even though the physicians’ accumulated experience in intracranial stenting may also play an important role in decreasing the risk of endovascular treatment.

### 3.2 Basilar artery stenosis

#### 3.2.1 Anatomy of the basilar artery

After arising from the unification of both vertebral arteries at the pontomedullary sulcus level, the basilar artery ascends along the pons within the basilar sulcus to the pontomesencephalic sulcus and bifurcates into two posterior cerebral arteries (Giotta Lucifero et al., 2021). The basilar artery gives rise to the anterior inferior cerebellar artery, pontine arteries, and superior cerebellar artery. Moreover, many perforating arterial branches are present in the basilar artery, especially in the middle segment. There are three groups of perforators (Marinkovic and Gibo, 1993): the caudal group has 2–5 perforators with a diameter ranging 80–600 microns, the middle group has 5–9 perforators with a diameter range of 210–940 microns, and the rostral perforators range 1–5 in number and 190–800 micorons in diameter and originate from the terminal part of the basilar artery (91.6 %), the superior cerebellar artery (91.6 %) or the posterolateral artery (16.6 %). Major complications of angioplasty and deployment of stents or flow diverting devices in the posterior circulation are caused by compromise of the basilar artery perforating branches (Lescher et al., 2014), and the small diameter of these perforators makes it difficult to be visualized by different angiographic imaging modalities, including computed tomography angiography, magnetic resonance imaging angiography or digital subtraction angiography.

#### 3.2.2 Treatment approaches

Basilar artery stenosis mostly involves the middle segment of the basilar artery which gives rise to the highest number of perforating arteries, resulting in clinically significant neurological deficits and contributing to a greater risk of peri-procedural complications (Kim et al., 2005, Bai et al., 2016). Because of the greater tortuosity and smaller caliber of the posterior circulation arteries, increased technical challenges of endovascular treatment may be encountered with significant risks of intervention-related complications (Jiang et al., 2010). The first-line treatment choice for basilar artery stenosis is aggressive medication combined with intensive control of risk factors, and for symptomatic refractory basilar artery stenosis, the second-line treatment options, including balloon angioplasty and stenting, have been applied. Elective PTAS is usually performed in patients with non-acute recurrent symptomatic posterior circulation infarction in spite of intensive medication because non-acute cases may have stable plaques and symptoms (Rasmussen, 2001). The cutoff values of the basilar artery stenosis for endovascular treatment varies across different medical centers, ranging from stenosis ≥ 50 % (Kim et al., 2005, Levy et al., 2003) to ≥ 70 % (Bai et al., 2016, Liu et al., 2016, Jia et al., 2017), which may be related to variable expertise and related caution in different medical centers with selection of patients who were mostly expected to achieve the most favorable outcomes of treatment.

Initially, angioplasty alone was used for endovascular treatment of basilar artery stenosis (Sundt et al., 1980, Sheikh et al., 2000, Hyodo et al., 1998), and later, angioplasty-assisted stenting was applied because of the proved lower rates of residual stenosis after stenting (Qureshi et al., 2008). Although use of balloon-expandable coronary stents had demonstrated higher stenosis decrease compared to angioplasty alone (Rasmussen, 2001, Gomez et al., 2000, Levy et al., 2002), the limited flexibility and requirement for great-pressure dilatation of these stents in deployment had posed some significant challenges in navigating through the tortuous posterior circulation and increasing the risk of iatrogenic arterial injuries. Introduction of the balloon-expandable Apollo stent and self-expandable Wingspan stent has improved the peri-procedural outcomes to some extents (Liu et al., 2016, Jia et al., 2017, Liu et al., 2020). Because the Apollo stent is stiffer and more difficult to navigate through tortuous arterial segments, it may be better suitable for straight Mori type-A stenosis. The strong radial force of this stent makes it optimal for stenosis with heavy calcification by direct stenting even with no predilatation angioplasty (Liu et al., 2020, Jiang et al., 2007). On the contrary, the Wingspan stent may be more adaptable to longer and tortuous Mori types B and C stenoses with no need of pre-stenting balloon dilatation (Jia et al., 2017, Samaniego et al., 2013). Use of antithrombotic treatment before and after endovascular intervention coupled with intra-procedural use of heparin may be able to significantly decrease the risk of instent thrombosis, which is required to balance with the potential concern of hemorrhagic complications.

### 3.3 Non-acute intracranial vertebrobasilar artery occlusion

Basilar artery occlusion takes up approximately 1 % of cerebral ischemic strokes and 10 % of large vessel occlusions, associating with high mortality and morbidity (Hu et al., 2022). Some patients may survive the acute stage but suffer from recurrent transient ischemic attacks and strokes at the chronic stage despite aggressive medical management (Caplan, 2012, Gorelick et al., 2008, Wang et al., 2014). Symptomatic chronic intracranial vertebrobasilar artery occlusion is frequently accompanied by insufficient collateral circulation for blood supply compensation, which may result in a high rate of ischemic events and disastrous outcomes refractory to best medication (Hawkes et al., 2020, Lindsberg et al., 2005). Recently, endovascular recanalization of chronic occlusion of intracranial arteries has been increasingly conducted, but the peri-procedural complications and mortality rates remain high (8.3 %-44.4 %) (Hawkes et al., 2020, Lindsberg et al., 2005, Ma et al., 2019a, Dashti et al., 2010, Aghaebrahim et al., 2014). Nonetheless, a retrospective one-center study investigating the natural history of basilar artery occlusion among 86 patients aged 60–79 years (13 or 17 % received intravenous thrombolysis, 21 or 25 % received endovascular treatment, and the rest 52 were treated with anticoagulation and/or antiplatelet therapy) even reported a higher mortality rate (Hawkes et al., 2020), with 34 % deaths during the initial hospitalization and only 41 % survivors at one year, which may indicate the necessity of active management for intracranial vertebrobasilar artery occlusion. Moreover, with accumulation of endovascular treatment experience, better skills have been obtained to ensure better outcomes for these patients. In a study exploring endovascular recanalization using the Enterprise stent for patients with symptomatic non-acute occlusion of intracranial vertebrobasilar artery occlusion with a duration from symptom onset to recanalization from 2 to 89 days (median 23 days) (Zhou et al., 2023a), the success recanalization rate was 92.0 %, and the peri-procedural complication rate was 19.3 % with a mortality rate of 5.7 %.

## 4 Peri-procedural complications of endovascular treatment

### 4.1 Stenosis of the posterior circulation

The peri-procedural complication rate of endovascular treatment of atherosclerotic stenosis is higher in the posterior than in the anterior circulation (Groschel et al., 2009, Nordmeyer et al., 2018). A meta-analysis enrolling 1177 patients with intracranial atherosclerotic stenosis treated with stent angioplasty reported a complication rate of 6.6 % and 12.1 % in the anterior and posterior circulation, respectively (Groschel et al., 2009). The peri-procedural ischemic event rate in the area of perforators was reported as 14.5 % in the posterior versus 5.1 % in the anterior circulation, and the recurrent stroke rate was 10.4 % in the posterior versus 3.4 % in anterior circulation (Nordmeyer et al., 2018). The higher complication rate caused by stent angioplasty in the posterior circulation may be associated with abundant perforators of the basilar artery, and balloon dilatation and stent delivery may generate a "snowplow effect" to cause perforator infarction (Li et al., 2015). Authors have reported a significantly lower complication rate of stent angioplasty in the intracranial vertebral artery stenosis than in the basilar artery stenosis (1.9 % vs. 7.5 %, P < 0.05) (Zhou et al., 2023a). These inherent features of the posterior circulation require strict adherence to relevant indications for patient selection, experienced operators to perform stent angioplasty, and softer and more flexible intracranial stents and delivery system to avoid injury to the arterial wall.

### 4.2 Non-acute occlusion of the posterior circulation

In revascularizing chronic occlusion of cerebral arteries, the commonest complications are arterial perforation and dissection (Kao et al., 2007), resulting in poor prognoses. In the study enrolling 9 patients with basilar artery occlusion treated endovascularly (Dashti et al., 2010), arterial dissection took place in 2 cases, stent thrombosis in 1, and arterial perforation in 1 during the peri-procedural period. In another study with 9 patients of chronic basilar artery occlusion (Aghaebrahim et al., 2014), endovascular recanalization caused arterial dissection in 1 case and perforation in 1, leading to severe disability in 1 patient and death in the other. The major reason for these severe complications is that stent angioplasty may injure important perforators of the basilar artery and cause severe cerebral function injury. In one study by Zhou et al. enrolling 88 patients with symptomatic chronic vertebrobasilar artery occlusion treated with endovascular revascularization (Zhou et al., 2023a), arterial dissection took place in seven (19.3 %) patients (5 cases with vertebral artery occlusion and 2 basilar artery occlusion). Two (2.27 %) patients with basilar artery dissection died probably because of injury to the basilar artery important functional perforators, whereas five (5.68 %) patients with intracranial vertebral artery dissection survived and had better prognosis probably because the vertebral artery does not have so many important perforators as the basilar artery does. In the study with 9 vertebral artery occlusion (Aghaebrahim et al., 2014), good prognosis (0 mRS in 1 patient and 1 in the other) was also reported in two patients with vertebral artery dissection.

## 5 Treatment outcomes of endovascular recanalization

### 5.1 Stenosis of the posterior circulation

Good outcomes may be obtained in endovascular treatment of basilar artery stenosis. In a systematic review and meta-analysis including 25 retrospective cohort studies comprising 1016 patients with symptomatic severe (≥50 %-70 %) basilar artery stenosis treated with angioplasty in 95.5 % of patients and/or stenting in 92.2 % (Palmisciano et al., 2023), the basilar artery stenosis was reduced from 81 % (53 %-99 %) to 13 % (0–75 %), successful intervention was 100 %, “good” final outcome was achieved in 89 % (95 % CI 85 %-93 %), intervention-related recurrent ischemic strokes were in 8.3 % (85 patients), and actuarial rates of intervention-related dissection, restenosis, and death were 0 %, 1 %, and 0 %, respectively. Long-term good prognosis with few recurrent strokes was also reported for patients with intracranial vertebrobasilar artery stenosis treated with endovascular recanalization (Djurdjevic et al., 2019). Among 30 patients with intracranial vertebral artery stenosis (n = 24) and basilar artery stenosis (n = 6) treated with endovascular recanalization (Djurdjevic et al., 2019), three patients (10 %) had recurrent strokes and two (6.7 %) experienced a transient ischemic attack in the posterior circulation at the follow-up of a median time of 7 years.

### 5.2 Non-acute occlusion of the posterior circulation

For symptomatic non-acute occlusion of vertebrobasilar artery, endovascular revascularization was found to be feasible even for a long segment of occlusion, with a high recanalization rate, a low complication rate, and good prognosis (Zhou et al., 2023a). In a study enrolling 88 patients with non-acute occlusion in the intracranial vertebral artery in 68 (77.27 %) patients and basilar artery in 20 (22.73 %) with an occlusion length of 4.5–43.7 mm (mean 18.3 ± 8.8) (Zhou et al., 2023a), endovascular revascularization was successful in 81 (92.0 %) patients, the residual stenosis was 10 %-40 % (mean 20.2 % ± 7.6 %), and periprocedural complications occurred in 17 (19.3 %) patients, with a mortality rate of 5.7 %. At follow-up of 79 (95.18 %) patients 5–29 (mean 16.9 ± 5.5) months later, the mRS score was significantly (P < 0.0001) better than that at discharge, stroke occurred in nine patients (11.4 %) at one year, and in-stent restenosis occurred in 19 (25.33 %) patients.

## 6 Hyperperfusion syndrome

### 6.1 Severity of the hyperpersufion syndrome

Because of poor perfusion and unstable hemodynamics caused by severe intracranial atherosclerotic stenosis, patients may have recurrent ischemic stroke, and timely elimination of the stenosis is necessary to prevent severe neurological deficits (Ma et al., 2019b, Nouh et al., 2014, Zhou et al., 2023b). Stent angioplasty is good for intracranial atherosclerotic stenosis both in the anterior and posterior circulations (Alexander et al., 2019, Alexander et al., 2021, Gao et al., 2022, Liu et al., 2016, Jia et al., 2022, Wang et al., 2022, Park et al., 2022, Guo et al., 2022, Zhou et al., 2019, Wang et al., 2020, Lin et al., 2021) because it can not only restore the stenotic lumen but also prevent recoil of the arterial wall following balloon dilation, helping maintaining the arterial lumen and prevent recurrent ischemic stroke. Nonetheless, hyperperfusion syndrome may occur as a potentially devastating postoperative complication following successful recanalization of severe intracranial arterial stenosis, possibly resulting in neurologic dysfunction, seizure, or reperfusion hemorrhage (Xu et al., 2015, Rezende et al., 2006, Ghuman et al., 2019, Yan et al., 2023). This syndrome is less well-investigated, but may take place in 3.4 % of patients with almost 80 % mortality when complicated by intracerebral bleeding, contributing to poor outcomes after stent angioplasty (Ghuman et al., 2019).

### 6.2 Measures to control the syndrome

Rigorous control of blood pressure is commonly applied to prevent the cerebral hyperperfusion syndrome in the peri-procedural period following stent angioplasty of severe intracranial stenosis. Failure to strictly control the blood pressure after recanalization, an interval less than 3 weeks between the recanalization procedure and presence of the last ischemic symptoms, and poor collateral circulation have been revealed to significantly associate with the development of hyperperfusion syndrome (Xu et al., 2015). Nitroprusside is not suggested for use to control blood pressure after the recanalization because it may cause considerable fluctuations of blood pressure (Xu et al., 2015). Nonetheless, optimal blood pressure control is frequently arbitrary, and different imaging biomarkers predictive of the hyperperfusion syndrome have been described. Angiographic signs of an early draining vein and capillary blush after endovascular recanalization have been suggested as sentinel signs for cerebral hyperperfusion, which may reflect the underlying impaired autoregulation of cerebral arteries (Ghuman et al., 2019). Early magnetic resonance imaging pseudo-continuous arterial spin labeling (PCASL) perfusion examination is able to document cerebral blood flow increase suggestive for cerebral hyperperfusion syndrome (Diana et al., 2021). A significantly higher volume of time-to-maximum (Tmax) > 4 s may be a predictor of the hyperperfusion syndrome after stent angioplasty in symptomatic intracranial arterial stenosis (Yan et al., 2023).

### 6.3 Risk factors for hyperperfusion syndrome

The hyperperfusion syndrome after stent angioplasty of the posterior circulation stenosis or occlusion has been less recorded than that in the anterior circulation (Meyers et al., 2006, Katsumata et al., 2021, Bando et al., 2001, Zhang et al., 2009). In posterior circulation stenosis, reduced cerebral blood flow in the posterior circulation before endovascular recanalization may be a risk factor for hyperperfusion syndrome following stenting (Katsumata et al., 2021). Signs and risk factors for the hyperperfusion syndrome in the treatment of anterior circulation stenosis can be used for reference of that in the posterior circulation. In carotid stenosis treated with stent angioplasty, age (≥70 years), severity of carotid artery stenosis (≥90 %), and poor collateral circulation have been significantly associated with a higher rate of the hyperperfusion syndrome (Xu et al., 2021), and patients with severe bilateral carotid stenoses may also be predisposed to the hyperperfusion syndrome, particularly when concurrent with arterial hypertension (Abou-Chebl et al., 2004). Severe unilateral carotid stenosis, presence of near-complete arterial occlusion, bad compensation of the Willis' Circle, and pre-recanalization time-to-peak index> 1.22 have also been reported as high risks of developing the hyperperfusion syndrome after carotid artery stenting.

## 7 Risk factors for prognosis of endovascular interventions

### 7.1 Stenosis of the posterior circulation

Because the basilar artery possesses many fine perforating branches, the risk of perforator strokes has to be balanced when enrolling appropriate patients for endovascular treatment. It has been found that patients with diabetes mellitus, pre-procedural stenosis < 88.4 %, and/or < 18 years from the last symptom onset to the procedure have significantly (P < 0.05) higher risks, possibly because of the “snowplow effect” caused by stent angioplasty on some potential unstable plaques to displace atheromatous debris over the perforators’ ostia (Jia et al., 2017). In applying the Enterprise stent for the treatment of severe symptomatic intracranial vertebrobasilar artery stenosis (Zhou et al., 2023a), coronary heart disease, diabetes mellitus, and stenotic site at the basilar artery were found to be significant (P < 0.05) risk factors for periprocedural complications. While stenosis length, plaque shape, and tirofiban use were demonstrated to be significant risk factors for the 1-year stroke or dearth rate, hyperhomocysteinemia and stenosis length were significant risk factors for in-stent restenosis. Nevertheless, the stenosis length was the only independent risk factor for the 1-year stroke or death rate and in-stent restenosis. As the stenosis length increases, a longer stent is required and may increase the risk of in-stent thrombosis, restenosis and recurrent stroke. In a study exploring the long-term recurrent stroke rate in symptomatic patients of intracranial atherosclerotic stenoses treated with stent angioplasty (Zhang et al., 2020), posterior circulation stenosis was found as an independent risk factor for recurrent stroke while good antegrade flow (TICI 3/4) was found to lower the recurrent risk in the posterior circulation stenosis after endovascular therapy.

### 7.2 Non-acute occlusion of the posterior circulation

For endovascular recanalization of non-acute occlusion of the intracranial vertebrobasilar artery, one study treated patients with the duration from symptom onset to endovascular recanalization of 2–89 (median 23) days (Zhou et al., 2023a). It reported blunt occlusion as an independent risk factor for successful revascularization, blunt occlusion and duration from symptom onset to recanalization as two independent risk factors for peri-procedural complications, and post-dilation as an independent risk factor for both in-stent restenosis and 1-year death or stroke events. Admission National Institutes of Health Stroke Scales (NIHSS) score, posterior circulation Alberta Stroke Program Early Computed Tomography Score (pc-ASPECTS), and successful recanalization were significant independent predictors of 90-day clinical outcomes after endovascular treatment of basilar artery occlusion (Hu et al., 2022, Novakovic-White et al., 2021, Alemseged et al., 2022). Good collateral circulation may be associated with a low mortality rate, and women have good collateral grades than men do (Hu et al., 2022, Chalos et al., 2019, Wiegers et al., 2020). Thus, sex may also be an independent predictor of mortality within 90 days as revealed by one study (Hu et al., 2022) even though some studies did not find the association of sex with clinical outcomes of endovascular treatment (Chalos et al., 2019, Tan et al., 2022). Estrogen has some neuroprotective and anti-inflammatory effects on the endothelial cells of cerebral arteries, and the risk of ischemic stroke may rapidly increase in postmenopausal women who experience decreased estrogen levels (Koellhoffer and McCullough, 2013).

Better operators’ expertise, experience, and device maneuverability may minimize iatrogenic movement of the basilar artery and prevent avulsion of perforators, thus helping decreasing perforators’ strokes (Liu et al., 2020, Tang et al., 2021).

## 8 Strategies for endovascular treatment of intracranial vertebrobasilar stenosis

### 8.1 Types of lesions and different stents

Balloon-expandable coronary stents initially used had limited flexibility to pass through the tortuous cerebral vasculature and required a greater pressure for dilation and deployment, which had increased the peri-procedural complications (Rasmussen, 2001, Gomez et al., 2000, Levy et al., 2002). Subsequent introduction of balloon-expandable Apollo stent and self-expandable Wingspan stent had improved the peri-procedural complications (Liu et al., 2016, Jia et al., 2017, Liu et al., 2020). Nonetheless, both the Apollo and Wingspan stents are stiffer, have strong radial force, and are difficult to traverse tortuous arterial segments, which predispose them to a relatively high peri-procedural complication rate in stent angioplasty of intracranial arterial stenosis (Zaidat et al., 2015, Liu et al., 2020, Jiang et al., 2007, Tsivgoulis et al., 2016, Fiorella et al., 2012). The Apollo stent may be better suited for straight Mori type-A stenosis with heavy calcification (Liu et al., 2020, Jiang et al., 2007) while the Wingspan stent may be better for longer and tortuous Mori types B and C stenoses (Jia et al., 2017, Samaniego et al., 2013). The tip of the Wingspan stent is hard and can easily produce arterial injury and subsequent post-operative restenosis (Fujimoto et al., 2013). In managing intracranial atherosclerotic stenosis, the Wingspan stent was associated with a higher peri-procedural ischemic rate in the posterior circulation versus in the anterior circulation (14.5 % vs. 5.1 %) and a greater recurrent stroke rate in the posterior circulation versus in the anterior circulation (10.4 % vs. 3.4 %) at follow-up 19 months later (Nordmeyer et al., 2018).

### 8.2 Rich perforators and flexible stents

Because of presence of rich perforators of the intracranial vertebrobasilar artery, flexible stents should be selected for stent angioplasty of arterial stenosis in this location so as to reduce the peri-procedural complications. In comparison with the Wingspan stent, the Enterprise stent and the Neuroform Atlas stent are both flexible and have a smaller radial force, beneficial in reducing the peri-procedural complications (Buonomo et al., 2021, Shofti et al., 2004). The closed-cell design of the Enterprise stent makes it easier to operate and deliver accurately via a soft flexible microcatheter through tortuous cerebral arteries (Zhou et al., 2023a). In a study investigating the Enterprise stent for the treatment of 209 intracranial atherosclerotic stenoses in 189 patients (Vajda et al., 2012), the technical success rate was reported to be 100 %, a major peri-procedural complication rate 8.1 %, a restenosis rate 24.7 % at 4.2-month follow-up, and a recurrent ischemic rate related to the stented artery 2.2 % at 10.2 months. In another study with 35 patients of severe symptomatic basilar atherosclerotic stenosis treated with the Enterprise stent angioplasty (Zhao et al., 2016), the technical success rate was reported to be 100 %, and a main procedural complication rate was 0, with a restenosis rate of 17.6 % at 6-month follow-up and a transient ischemic attack rate of 5.7 % at 10.6-month follow-up. In a study investigating the Enterprise stent angioplasty for 275 intracranial vertebrobasilar stenotic lesions in 262 patients (Zhou et al., 2023a), the technical success rate was 100 %, complications occurred in 14 (5.3 %) patients, recurrent strokes took place in 7 patients (2.9 %) at 1-year follow-up, in-stent restenosis was present in 35 patients (15.7 %), with a restenosis rate of 26.4 % (n = 23) in the intracranial vertebral artery, which was significantly (P < 0.001) greater than in the basilar artery (8.8 %). The Neuroform Atlas stent is of a hybrid design with alternation of closed and open cells and can be deployed in an artery with a diameter of 2–4.5 mm. Limited study outcomes were encouraging using the Neuroform Atlas stent for the treatment of symptomatic intracranial stenosis as an off-label application (Buonomo et al., 2021).

### 8.3 Recanalizaiton extent

When recanalizing the intracranial atherosclerotic stenosis, sub-satisfactory recanalization may be a better strategy, which does not require complete restoration of the stenotic lumen to the normal arterial diameter. Rather, sub-satisfactory recanalization is referred to stenting recanalization of arterial stenosis with a balloon whose diameter was less than 80 % of the nearby normal arterial diameter for expanding the stenosis prior to deployment of a stent with a residual stenosis less than 30 %. This sub-satisfactory recanalization is able to restore the vascular stenosis, prevent recoil of the arterial wall, decrease possible peri-procedural ocmplications, and prevent possible hyperperfusion syndrome (Zhou et al., 2023b, Zhang et al., 2022). Complete restoration of the stenotic lumen may require a greater pressure for dilation and consequently crush unstable atherosclerotic plaques with production of debris to occlude arterial branches and subsequent ischemic stroke, and use of a hard stent with a large radial force may also require a greater pressure for expanding the artery, resulting in a similar outcome. This strategy has produced some positive outcomes in one study investigating the safety and efficacy of the Enterprise stent for angioplasty of symptomatic severe (≥70 %) posterior circulation stenosis (Zhou et al., 2023a). In this study (Zhou et al., 2023a), sub-satisfactory stenting recanalization resulted in a reduction of the stenosis from 86.3 ± 6.2 % before to 19.3 ± 5.4 % after stenting, a peri-procedural complication rate 5.3 %, a 1-year recurrent stroke rate 2.9 %, and a follow-up in-stent restenosis rate 15.7 %. This was similar to the outcome of the CASSISS study investigating the effect of the Wingspan stenting plus medical therapy vs. medical therapy alone on risk of stroke and death in patients with symptomatic intracranial stenosis, which obtained a complication rate of 5.1 % and a 1-year recurrent stroke rate of 2.8 % (Gao et al., 2022). Another study exploring the Enterprise stent in endovascular treatment of intracranial atherosclerotic arterial stenosis also revealed a major procedural complication rate 8.1 %, a 30-day combined neurological morbidity and mortality rate 0.9 %, a restenosis (>50 %) rate 24.7 % 4.2 months later, and an incidence of recurrent ischemia related to the stented artery 2.2 % during 10.2 months of mean follow-up (Vajda et al., 2012).

## 9 Evidence of reperfusion improvement after endovascular treatment

Sub-satisfactory recanalization of severe middle cerebral arterial stenosis has been reported to significantly improve the hemodynamic parameters of wall shear stress, total pressure, velocity and vorticity proximal or distal to the stenosis and at the perforator after computational fluid dynamic analysis of the hemodynamic stresses (Zhang et al., 2022). After sub-satisfactory stenting recanalization, all hemodynamic parameters including blood perfusion pressure (total pressure) and flow velocity were improved to be closer to those after virtual restoration of the arterial lumen to nearly the normal diameter. After investigation of changes in the hemodynamic parameters following sub-satisfactory stenting recanalization of basilar artery stenosis (Zhou et al., 2023b), Zhou et al. reported that after significant restoration of the basilar artery stenosis by sub-satisfactory recanalization, the hemodynamic parameters near the stenosis and at a perforator have been significantly improved, including the total blood perfusion pressure, wall shear stress, cell Reynolds number, flow velocity, vorticity, turbulence intensity, turbulence kinetic energy and dissipation rate, thus significantly improving the cerebral perfusion pressure and flow velocity, similar to those changes after virtual restoration of the stenotic arterial lumen to nearly the normal arterial diameter.

## 10 Conclusion

Cerebral arteries of the posterior circulation are tortuous and complex and may pose some challenges for angioplasty and stent deployment in the treatment of basilar atherosclerotic stenosis, and many perforating branches of the basilar artery may increase the peri-procedural complications of endovascular recanalization. Nonetheless, PTAS may be feasible, effective and safe in selected patients with symptomatic severe (≥ 50 %-70 %) stenosis and non-acute occlusion refractory to the best medical management. The treatment approaches and endovascular devices should be tailored to the clinic-radiological features of each lesion and patient. Sub-satisfactory stenting recanalization of the stenosis using a balloon < 80 % of the diameter of the nearby normal artery for dilatation of the stenosis and selection of softer and more flexible stents and delivery systems for deployment may be advantageous in decreasing the peri-procedural complications.

## Funding

This study was supported by the Research and Promotion Project on Appropriate Intervention Technology for Stroke High-Risk Groups in China (GN-2018R0007) and the 13th Five‑year Plan of China for Research and Development (2016YFC1300702).

## Author contributions

LF Z, TX L, and BL G contributed to the conception and design of the study; ZL Z, LF Z, TX L, and BL G contributed to the acquisition and analysis of data; ZL Z, LF Z, TX L, and BL G contributed to drafting the text.

## CRediT authorship contribution statement

**Zhi-Long Zhou:** Visualization, Validation, Supervision, Investigation, Formal analysis, Data curation. **Liang-Fu Zhu:** Visualization, Validation, Supervision, Project administration, Methodology, Data curation, Conceptualization. **Tian-Xiao Li:** Visualization, Validation, Supervision, Investigation, Data curation. **Bu-Lang Gao:** Writing – review & editing, Writing – original draft, Visualization, Validation, Investigation, Data curation.

## Declaration of Competing Interest

None.
